# Specialty Grand Challenge in Veterinary Surgery and Anesthesiology

**DOI:** 10.3389/fvets.2015.00019

**Published:** 2015-07-17

**Authors:** Christopher R. Byron

**Affiliations:** ^1^Department of Large Animal Clinical Sciences, Virginia-Maryland College of Veterinary Medicine, Blacksburg, VA, USA

**Keywords:** surgery, anesthesiology, education, One Health, evidence-based medicine, reporting guidelines, CONSORT, ARRIVE

## Introduction

Truly incredible advances have been gained in the past few decades with regard to the clinical practice of veterinary surgery and anesthesiology. Novel investigational techniques and medical procedures have greatly enhanced our abilities to diagnose and treat animals. Use of minimally invasive diagnostic and procedural techniques, improvements in medical equipment, and the advent of molecular biological methods have advanced veterinary medical practice to a level well beyond what was possible just a few short years ago. However, tough challenges remain that will require novel approaches and new ways of thinking. Education of future clinician scientists is at the heart of this advancement; traditional instructional techniques may not meet the needs of future students. An increase in the flexibility and efficiency of veterinary medical education will be needed. In addition, we must have greater awareness of the interplay between human, animal, and environmental health. The importance of these relationships has recently been identified in the tenets of the One Health initiative; this movement has great relevance to the advancement of veterinary surgery and anesthesiology. Another critical component in the growth of these specialties will be concerted application of evidence-based clinical decision making. To that end, we will need to deliberately design high-quality randomized controlled clinical trials and to report the findings of these studies transparently and completely. In this editorial, I will make the case for the importance of these issues in veterinary surgery and anesthesiology and for the role of this specialty journal section in solving these problems. The purpose of the *Veterinary Surgery and Anesthesiology* section of *Frontiers in Veterinary Science* is to provide an efficient forum for dissemination and discussion of high-quality scientific information relevant to these and closely related disciplines. This is an exciting endeavor because it fosters engagement of authors, peer reviewers, editors, and readers in the process. Together, we can tackle these challenges!

## A Paradigm Shift in Veterinary Surgery and Anesthesia Education

Veterinary education is presently facing many simultaneous challenges. The costs of education for students are rising dramatically and, compounding this problem, recent economic analysis indicates that the net present value and return on investment of veterinary education are low (1). To address these and other issues facing the veterinary educational community, the North American Veterinary Medical Education Consortium determined strategic goals and recommendations including graduation of proficient career-ready veterinarians, use of competency-driven assessments, sharing of resources within the veterinary education community, increasing the economic viability of veterinary education, and increasing focus on innovation (2). Although there appears to be wide support for such measures, progress is slow and will require ongoing efforts (3).

Along with veterinary medical education in general, there has been a paradigm shift in veterinary surgery and anesthesia education. Trends are toward decreased use of live animals for teaching of procedures. As a result, there is great interest in use of alternate educational methods. Dedicated clinical skills laboratories are becoming more common at veterinary teaching institutions; these facilities incorporate innovative methods and custom models for use in surgical instruction. Validation is an important component of the process for refinement of these teaching methods. The goal is to improve the surgical education of veterinary students, interns, and surgical residents while decreasing unnecessary use of live animals. Recently, development of simulators (4) and use of video games (5) has been investigated for instruction and prediction of minimally invasive surgery skills of veterinarians and veterinary students. In addition, minimally invasive surgery can be useful for the instruction of traditional clinical skills. As an example, the use of laparoscopic guidance can improve student learning of rectal examination in horses (6). Such approaches are likely to become more common in traditional and minimally invasive surgical education. It is critical that descriptions and results of scientific analysis of these techniques be disseminated through primary literature publications. Use of scientific methodology for analysis of pedagogical approaches will result in improved educational methods through better and more efficient use of resources. Another benefit of this approach may be reduced cost of education, which will be increasingly important for economical delivery of instruction.

In addition to veterinary clinical education needs, there will be a growing requirement for training of scientists in relevant applied and basic science fields. With the pool of funding sources for support of scientific inquiry shrinking, discovery and training will continue to be a challenge. As well, recruitment of talented young clinician-scientists is difficult. New approaches to veterinary scientific exploration may increase available funding. In particular, more concerted efforts should be made to identify and evaluate naturally occurring animal diseases as models of human disease. In addition, veterinary scientific discovery would benefit from increased use of multidisciplinary and interdisciplinary teams of investigators working in multiple areas including veterinary and human clinical sciences, basic and applied biological sciences, chemistry, and materials engineering. Communication of such approaches to investigation through scientific journals is important, because areas in which further investigation is needed can be identified and such multidisciplinary approaches promoted.

Scientific journals play an integral role in supporting and advancing veterinary education. Peer review and dissemination of the descriptions of pedagogical techniques should be encouraged. Similarly, the efficacy of competency-driven assessments of skills (including surgery and anesthesia) will require validation. High-quality open-access publications are an efficient and cost-effective avenue for delivery of information to students and instructors. Description and validation of novel techniques that reduce the use of live animals in instruction and increase the economic and educational efficiency of veterinary learning can be effectively accomplished through publication in such a forum. And, journals such as Frontiers provide a high-quality open-access mechanism for dissemination of peer-reviewed manuscripts that are essential for the support of veterinary graduate students and investigators.

## One Health: Veterinary Surgical Models of Human Disease

The above approaches to invigorating veterinary scientific education and discovery overlap with several tenets of the One Health Initiative. This movement is intended to foster co-equal collaborations between veterinary and human medical professionals to enhance the health of animals, humans, and the environment^1^. Veterinarians are uniquely positioned to contribute to these goals^2^. Prominent examples of topics for which this movement is expected to have high impact are zoonotic and vector-borne diseases, food production, the human–animal bond, and population growth. In the disciplines of veterinary surgery and anesthesiology, diagnosis and treatment of naturally occurring diseases often have direct applicability to One Health. Many animal diseases are excellent models of human disease; techniques for treatment of diseases in veterinary species can substantially aid treatment of diseases in humans, and vice versa. For example, femoropatellar joint osteoarthritis is a common malady that develops spontaneously in dogs. Experimentally induced and naturally occurring knee arthritis in dogs are considered excellent models of such diseases in humans (7) because diagnostic techniques, treatment methods, and outcome measures are similar in these species. Therefore, advances in osteoarthritis treatment for either humans or dogs can be of direct benefit to the other species. Horses are another veterinary species that commonly develop degenerative joint disease. Osteoarthritis is the most common cause of lameness in horses. Therefore, this is a potentially useful model for osteoarthritis in humans. As with dogs, the diagnostic methods and medical and surgical treatments for horses with arthritis are very similar to those used for humans. In addition, veterinarians have extensive clinical experience with this disease in horses (8). Such experiences, together with the commonality of the disease, afford the opportunity to use knowledge of equine osteoarthritis pathophysiology, biomarker detection, targets of therapy, and outcomes for the purposes of comparative rheumatology. Reliable experimental induction of osteoarthritis in horses, including use of non-terminal models (9), can also contribute greatly to such comparative approaches to human disease.

Recognition of animal diseases as models of human diseases is vitally important. However, an efficient method is needed to allow timely sharing of basic and clinical scientific knowledge. To that end, internet-based registries of diseases can be an effective way to disseminate such data; this could encourage multi-institutional studies, potentially increasing numbers of animals in such studies and broadening the geographic relevance of such work. Finally, a critical component of this comparative approach to modern medical advancement is an efficient scientific communication method. Expedited communication of high-quality scientific discovery via open-access journals, such as Frontiers in Veterinary Science, is an efficient mechanism to support the progress of investigators in these endeavors.

## Evidence-Based Veterinary Surgery and Anesthesia

Evidence-based medicine seeks to apply evidence determined via use of the scientific method for optimization of clinical decision making. There have been calls for deliberate and purposeful application of this principle in veterinary practice (10). Success will depend on a transition from reliance on personal clinical experience and published small case series to use of evidence from large randomized controlled clinical trials. Unfortunately, the available scientific evidence in veterinary clinical disciplines is often of low quality and insufficient to inform clinical decision making with confidence (11). The strength of such evidence is a continuum from results determined through *in vitro* studies or statements of personal opinion (low quality) to meta-analyses of large randomized controlled trials (high quality). Historically, impediments to achievement of this gold standard have hindered application of evidence-based medicine to veterinary surgery and anesthesiology. In particular, low case numbers in published studies from single institutions, the lack of well-defined outcome measures, and absence of appropriate controls diminish the quality of evidence and its applicability to a geographically diverse veterinary practice. Increased multi-institutional collaborations among investigators would lead to an increase in the number of animals enrolled in clinical trials and may also increase the relevance of results to a geographically diverse population. In addition, such studies should be thoughtfully designed to include biologically relevant outcome measures and, when possible, this information should be collected by use of validated clinical outcome instruments (12, 13). These outcome instruments include questionnaires, standardized clinical scoring algorithms, and objective measures, such as force plate data (for assessment of lameness), physiologic parameters, and biomarkers. Further, study designs should include appropriate control populations and incorporate sound statistical methods. An additional method for enhancing collaboration in clinical scientific discovery and increasing the statistical power of clinical trials may be use of online registry databases as mentioned above, which could greatly aid the speed and quality of discovery.

Scientific publications have a central role in evidence-based medicine. Dissemination of clinical evidence determined through well designed and carefully conducted studies provide the necessary scientific information for evidence-based decision making in veterinary practice. A high quality of manuscript preparation and meticulous review are vital in this process. Further, expedited and open-access publication ensure that clinically relevant evidence is disseminated in a timely fashion to the audience that most needs the information, regardless of their access to well-stocked medical libraries.

## Reporting Guidelines

An essential component of evidence-based medicine is meticulous reporting of the results of studies. Without this, findings cannot be adequately analyzed and considered by readers and therefore cannot be used for sound clinical decision making. Incomplete or inaccurate reporting of otherwise well-designed clinical trials therefore minimizes the impact of such important scientific knowledge. This can be a particularly vexing problem for veterinary surgery, as it is often difficult to accurately gage success and compare outcomes for various procedures because of sparse or incomplete information in the scientific literature. Likewise, these problems pose great difficulties for anesthesiologists; anesthetic complications for veterinary patients can be rare although important, and a lack of accurate and detailed reporting of clinical studies hampers use of such information to improve the quality and safety of patient care. Undoubtedly, shortcomings in reporting are typically unintentional and the result of the myriad choices that authors face when preparing manuscripts. Recently, there has been an effort to develop guidelines for standardized reporting of medical research. The EQUATOR network^3^ is an organization of medical research stakeholders including investigators, journal editors, and peer reviewers that have developed reporting guidelines for over 250 specific types of studies. The stated mission of this group is to “achieve accurate, complete, and transparent reporting of all health research studies to support research reproducibility and usefulness … to minimize avoidable waste of financial and human investments in health research projects.” One of the most relevant of these guidelines to surgery and anesthesiology is the CONSORT (Consolidated Standards of Reporting Trials) statement^4^. This is an evidence-based list of recommendations for reporting of randomized trials, and includes checklists and flow diagrams to help authors with complete and transparent reporting of study findings. Employment of these items substantially increases the quality and utility of published work. Important components include recommendations regarding descriptions of trial design, subject recruitment, outcomes, sample size, blinding, statistical methods, study limitations, and interpretation. In addition, the ARRIVE (Animal Research: Reporting of *In Vivo* Experiments) guidelines^5^ have been developed “to improve the design, analysis, and reporting of research using animals – maximizing information published and minimizing unnecessary studies.” These recommendations are applicable to all areas of animal research, and provide valuable resources that can readily be used to improve reporting of *in vivo* surgery and anesthesiology research. Deliberate use of standardized guidelines during study design, manuscript preparation, and peer review should be encouraged, as it ultimately enhances study quality and serves to maximize impact for evidence-based decision making.

## Summary

There are many important challenges facing the veterinary profession. In particular, challenges in educational methods, application of the tenets of One Health, integration of evidence-based medicine, and careful integration of standardized study design and reporting guidelines are germane to the specialties of surgery and anesthesiology. The *Veterinary Surgery and Anesthesiology* specialty section of *Frontiers in Veterinary Science* will be an excellent forum for rapid dissemination of high-quality research findings in many facets of our profession. As well, it will be an open forum for discussion of the challenges facing us. As per the mission statement, the goals of this section are dissemination of findings from clinical research and basic science studies of naturally occurring and experimental disease with translational potential. The use of multidisciplinary and interdisciplinary approaches is encouraged. It is my sincere belief that this will be a dynamic and effective forum for solving these and other challenges that lie ahead. I invite you to actively engage in this scientific discussion and help forge the future of veterinary surgery and anesthesiology!

## Conflict of Interest Statement

The author declares that the research was conducted in the absence of any commercial or financial relationships that could be construed as a potential conflict of interest.

## Footnotes

^1^http://www.onehealthinitiative.com

^2^www.avma.org/KB/Resources/Reference/Pages/One-Health94.aspx

^3^http://www.equator-network.org

^4^http://www.consort-statement.org

^5^https://www.nc3rs.org.uk/arrive-guidelines
